# The Organization of the Quorum Sensing *luxI/R* Family Genes in *Burkholderia*

**DOI:** 10.3390/ijms140713727

**Published:** 2013-07-02

**Authors:** Kumari Sonal Choudhary, Sanjarbek Hudaiberdiev, Zsolt Gelencsér, Bruna Gonçalves Coutinho, Vittorio Venturi, Sándor Pongor

**Affiliations:** 1 International Centre for Genetic Engineering and Biotechnology (ICGEB), Padriciano 99, Trieste 32149, Italy; E-Mails: sonal.kumari@icgeb.org (K.S.C.); sanjarbek.hudaiberdiev@icgeb.org (S.H.); Bruna.Coutinho@icgeb.org (B.G.C.); 2 Faculty of Information Technology, PázmányPéter Catholic University, Práter u. 50/a, Budapest 1083, Hungary; E-Mail: gelzsolt@gmail.com; 3 The Capes Foundation, Ministry of Education of Brazil, Cx postal 250, Brasilia, DF 70.040-020, Brazil

**Keywords:** quorum sensing, *N*-AHL, local topologies, chromosomal arrangements, *Burkholderia*

## Abstract

Members of the *Burkholderia* genus of Proteobacteria are capable of living freely in the environment and can also colonize human, animal and plant hosts. Certain members are considered to be clinically important from both medical and veterinary perspectives and furthermore may be important modulators of the rhizosphere. Quorum sensing via *N*-acyl homoserine lactone signals (AHL QS) is present in almost all *Burkholderia* species and is thought to play important roles in lifestyle changes such as colonization and niche invasion. Here we present a census of AHL QS genes retrieved from public databases and indicate that the local arrangement (topology) of QS genes, their location within chromosomes and their gene neighborhoods show characteristic patterns that differ between the known *Burkholderia* clades. In sequence phylogenies, AHL QS genes seem to cluster according to the local gene topology rather than according to the species, which suggests that the basic topology types were present prior to the appearance of current *Burkholderia* species. The data are available at http://net.icgeb.org/burkholderia/.

## 1. Introduction

The *Burkholderia* genus are gram negative bacteria that are ubiquitous in the environment and may cause a number of diseases in plants, animals and humans. Opportunistic human infection can be particularly serious for patients with cystic fibrosis while other members are causative agents in various diseases. Conversely, *Burkholderia* are involved in nitrogen fixation in the rhizosphere and various species have the ability to metabolize pesticides and polychlorinated biphenyls (PCBs), making them attractive from an agricultural perspective (although in this latter case concerns have been raised because of the pathogenic potential of some species).

One of the most important aspects of understanding this genus as a pathogen or as beneficial microorganisms is that quorum sensing (QS) systems operating in *Burkholderia* are important regulators of virulence and other phenotypic traits. Furthermore, QS systems appear to be of crucial importance in governing overall colonization and niche invasion. This suggests that modulation of these regulatory systems might be an attractive route for controlling virulence and hence the development of interventions designed to treat infection or (in the case of agriculture) induce beneficial activities. It is therefore crucial that we have a more complete understanding of QS systems in *Burkholderia* and in particular the genetic basis upon which it operates.

One of the challenges faced by researchers is that *Burkholderia* are very diverse from taxonomic and genetic perspectives. For many years, *Burkholderia* were recognized as members of the non-fluorescent pseudomonads. However, pseudomonads were found to be taxonomically heterogeneous and five species homology groups were devised on the basis of rRNA-DNA hybridization experiments [1] and the genus *Pseudomonas* was divided into five well-defined rRNA homology groups. Subsequently, polyphasic taxonomy analyses, including 16S rRNA sequence analysis, DNA-DNA hybridization and fatty acid analysis, led to the establishment of the *Burkholderia* genus to accommodate seven species of the *Pseudomonas* rRNA group II (*P. cepacia*, *P. caryophylli*, *P. gladioli*, *P. mallei*, *P. pseudomallei*, *P. solanacearum* and *P. picketti)* [2,3]. *P. solanacearum* and *P. picketti* were later renamed as *Ralstonia*.

In the early 1980s, strains of *B. cepacia* were increasingly being recovered from cultures of respiratory tract specimens from cystic fibrosis patients [4]. Continuous taxonomic studies allowed the designation of binomial species names of clinically-isolated *Burkholderia* and are now referred to as the species of the *Burkholderia cepacia* complex (BCC) [5–7]. Given their clinical importance, the BCC members as well as other pathogenic *Burkholderia* such as *B. mallei* and *B. pseudomallei* have been the subject of intense analysis in terms of both their taxonomy and pathogenicity [8–12]. *B. pseudomallei* is for example the causative agent of melioidosis, an infectious disease endemic in southeast Asia and northern Australia (and may occur in other tropical and subtropical regions—in 1969, several cases of melioidosis were reported in non-human primates in the USA [13]). *B. mallei*, on the other hand is the causative agent of glanders in horses and is generally transmitted to humans through infected animals. *B. mallei* was also used as a bio-weapon in civil wars of America and Afghanistan [14,15]. Recently, other *Burkholderia* species have been characterized as potentially beneficial in terms of their role in plant-growth-promotion and nitrogen fixation in the rhizosphere and/or endosphere—many of them are diazotrophs and a few are capable of nodulation in mimosa and legumes [16–18].

Although their taxonomy is continually under review, the *Burkholderia* genus currently comprises over 60 validly described species. Phylogenetic trees inferred from independent gene sequence analyses (16S rRNA, *recA*, *gyrB*, *rpoB*, *acdS*), indicate that there are numerous divisions inside the *Burkholderia* genus with significant (>90%) bootstrap values [19–23]. Altogether, these analyses suggest that three major clades may exist inside the *Burkholderia* cluster. These include the *B. cepacia* complex (BCC), the “*pseudomallei*” group and a recently described clade that contains non-pathogenic plant-beneficial *Burkholderia* species.

Despite the apparent genetic diversity within the *Burkholderia* genus, a common characteristic is that they may utilize QS as part of their colonization and invasion strategies. QS is a process in which bacteria regulate gene expression according to their local population density. In gram-negative bacteria, the most common QS system involves the production and response to *N*-acylated homoserine lactones (AHLs) [24]. AHL production is catalyzed by an AHL synthase belonging to the LuxI-family of proteins; this enzyme requires *S*-adenosylmethionine (SAM) and an acylated acyl carrier protein (ACP) from the fatty acid biosynthesis pathway, as substrates. Upon reaching a minimal threshold level, AHL binds to a sensor/regulator protein that typically belongs to the LuxR-family of proteins. The *N*-terminal region of the LuxR protein has an AHL-binding domain which is reported to facilitate the formation of functional homodimers that allow it to bind to various DNA sequences and regulate the transcription of target genes [25]. Most commonly, one of the targets of the LuxR/AHL complex is the *luxI* gene thus creating a positive feedback loop which increases the production of AHL. This loop is important for the timing of the QS response in accordance with the AHL concentration and hence, population density [26].

Previous studies have shown that LuxR regulators are sometimes present in excess in comparison to AHL synthases. These have been termed as orphan or solo LuxR as they are not associated with cognate AHL synthases [27]. In some of the cases, these orphan or solo LuxR have been found to respond to endogenously/exogenously produced AHLs or to molecules produced by eukaryotes. These solo LuxR therefore, might help in the expansion of QS network and be involved in interspecies and inter-kingdom signaling [28,29].

According to an excellent review published in 2006, AHL QS circuitry is widely present in the genus *Burkholderia* [30]. In the present work we update this catalogue and provide new details on local gene arrangements, chromosomal locations, regulatory patterns and the chemical nature of QS signals distributed among the known and emerging sub-groups of the genus.

## 2. Local Topological Arrangement of Quorum Sensing Genes

One of the key aspects we are interested in, is the local topology of QS genes. This concept denotes the local arrangement of QS genes with respect to each other. The local arrangements found in Proteobacteria can be classified into 16 topology groups [31]. Denoting *luxI* genes as I, *luxR* genes as R, we can define a number of simple topologies, such as tandem orientation (*R⃑I⃑*), convergent orientation (*R⃑I⃐*), divergent orientation (*R⃐I⃑*), *etc*. In some more complicated arrangements, there is one intervening gene between the R and I genes, and this intervening gene frequently encodes a negative regulator [31]. The *rsaL* [32–34] and the *rsaM* [31,35,36] genes/homologs, denoted here by L and M, respectively, are typical examples. There are further arrangements which we term complex topologies [31] in which there are several intervening genes. A further classification is based on the position of the AHL system with respect to the replication origin within the chromosome, or alternatively on which chromosome the AHL system is located.

In addition to arrangement patterns, AHL systems can also be regarded as regulatory circuits that display a number of simple regulatory architectures [24,26,37]. The core element is the auto-inductive circuit of R and I genes that was originally used to define the concept of QS regulation [37]. However, in the absence of additional, stabilizing elements, autoinduction would raise signal levels without limit. A down-regulation loop activated at higher signal concentrations is perhaps the simplest way to limit and stabilize the signal levels. There are a variety of mechanisms that can play this stabilizing role in QS systems. For example, the master regulator TraM can form a non-functional heterodimer with the TraR gene product in *Agrobacterium tumefaciens* [38,39]. A homodimer of the RsaL gene product (denoted here as L) acts as negative regulator by binding on the bi-directional *rsaL-luxI* promoter [40]. RsaM (denoted here as M) is another small regulatory protein that might be acting in a similar way to RsaL in many *Burkholderia* species [35]. Bacterial genomes also encode enzymes that degrade AHLs in response to the stress signal ppGpp, which can also efficiently down-regulate QS signaling [41–45]. Down-regulation is also thought to result from more sophisticated, RNA-mediated mechanisms [46,47] or from the clash of transcriptional machineries on overlapping genes [48]. The crucial nature of these negative regulatory effects is shown by the fact that their deletion leads to signal overproduction and a less virulent bacterial phenotype. For the sake of completeness we also highlight that downregulation can also be automatically achieved by simple resource limitation where a reduction in number of QS cells would result in a decrease of the QS signal concentration itself [31].

Finally, yet another classification of AHL systems is possible on the basis of the chemical nature of the AHL signals they produce. It is well known that proteobacteria produce over 20 kinds of AHL signals [49,50], although much less are produced by selected genera such as *Pseudomonas* [51].

When this article was written (data collection was finished on 18 March, 2013), the NCBI archive contained 35 complete *Burkholderia* genomes, 57 draft genomes (30323 contigs and 266 scaffolds) and 16585 individual Genbank entries with *Burkholderia* sequences, a small portion of which contained full or partial AHL systems. Local gene arrangements were extracted from complete, well-annotated genomes, even though we noticed that the annotation of QS genes is at times inaccurate. Draft genomes contained some annotated genes, but frequently only the contig sequences were given. Finally, individual DNA sequences in GenBank rarely contained a complete AHL system, meaning that topological data could not be abstracted. In order to cope with these uncertainties, we re-annotated all DNA sequences using a sensitive computational procedure (described in Supplementary material). As for the other types of molecular data, regulatory circuit architectures and the chemical types of the AHLs produced did not form standard parts of databases; these had to be retrieved manually, as best we could, from the experimental papers. The result is an eclectic ensemble of data, some of it well annotated and reliable, some of it less well annotated, and inevitably, some of the research papers containing regulatory architectures or chemical data on AHL signals which may have gone unnoticed. For this reason, we primarily based our classification on validated data (such as complete genomes, review articles). A typical example of the difficulties is the classification of *Burkholderia* species based on 16S rRNA data. These data are available for complete genomes and have previously been used to define three main groups of *Burkholderia*: the *B. cepacia* complex, the *B. pseudomallei* group and the plant-beneficial and environmental (PBE) groups [52]. In addition there are further emerging groups that are defined by their hosts and pathogenicities, such as those of fungal symbionts [52] of plant-pathogenic species that may be partly overlapping with these groups. However, 16S rRNA data are not available for many of the newly discovered species.

## 3. A Brief Overview of AHL Systems in *Burkholderia*

A comparison of *Burkholderia* 16S rRNA sequences is known to yield three main taxonomical clusters/clades, *i.e*. the *B. cepacia* complex (BCC), the *B. pseudomallei* group (consisting of *B. mallei* and *B. pseudomallei*) and the recently defined plant-beneficial (PBE) group [52]. Of the near 20 AHL signals described so far (mainly C4 to C18-homoserine lactones (HSL) as well as HSLs with the C3 position substituted or unsubstituted by an oxo, or hydroxyl group), *Burkholderia* responds to six types of AHLs (Table 1).

On the other hand, the topological arrangements of the AHL systems within the *Burkholderia* genus show a broad variation. Briefly, *Burkholderia* species contain one to three AHL systems that follow different topological arrangements, as summarized in Table 2.

Out of the 16 local topological arrangements described in proteobacteria, 10 are found in *Burkholderia* (comprising of completely sequenced genomes, draft genomes and individual Genbank entries), including a new topology type named here as M4 (*R⃐M⃑X* (< 7)*I⃑*), which had previously not been detected in Proteobacteria. Out of 35 completely sequenced *Burkholderia* genomes, 32 (91%) were found to have AHL QS systems that belonged to eight distinct local topology types. In contrast, out of 44 completely sequenced genomes of *Pseudomonads*, only 11 (25%) had AHL QS genes that belonged to three topology types. Two of the three topology types, R1 (*R⃑I⃑*) and L1 (*R⃑L⃐I⃑*) were also present in *Burkholderia*. While the arrangements of AHL genes of pseudomonads are apparently simpler and have less intervening sequences between the R and I genes, the *Burkholderia* genus, especially the *B. pseudomallei* group, has a number of more complex arrangements.

The cladograms of LuxR and LuxI proteins show a seemingly complex classification scheme which can however be explained by the local topology of their AHL systems (Supplementary material). Namely, the I and R genes seem to classify according to their local topology, rather than according to the species. For instance, an I gene with a given topology within a species (e.g., *R⃑I⃑* in *B. pseudomallei* 1710b) is consistently more similar to an I gene of identical topology within another species (*R⃑I⃑* in *B.thailandensis* E264), than to an I gene of the same species, but having a different local topology (e.g., *R⃐M⃑I⃑* in *B. pseudomallei* ). It thus appears that local topology within the chromosome may have evolved before the separation of various *Burkholderia* clades.

**Figure 1:**
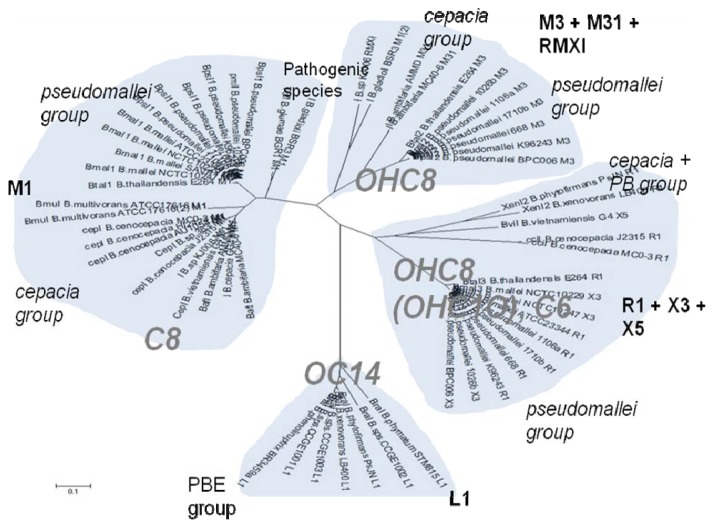
Clustering of LuxI protein sequences and perceived signals by LuxR homologues in complete *Burkholderia* genomes. In the following parts we review the AHL systems in the major *Burkholderia* clades.

As schematically shown in Table 3 and Figure 1, the chemical structure of the AHL signals is in good correlation with the local topology of the AHL systems, *i.e*., the same chemical signal is produced by AHL systems belonging to identical or related local topologies, or to a few topology types. The agreement across genera is less equivocal (details not shown). For instance, an AHL system of L1 topology in *B. xenovorans* is regulated by the signal OC14, while an analogous AHL system in *P. aeruginosa* is regulated by a different signal, OC12. In comparison, the R1 topology is connected to a variety of signals in at least 96 proteobacterial species in which it has been detected so far [31].

### 3.1. *Burkholderia cepacia* Complex (BCC)

The taxonomy of the *Burkholderia cepacia* complex refers to closely related species (previously called genomovars) that have been isolated from clinical samples and are opportunistic human pathogens. BCC is composed of at least 17 species, including *Burkholderia cepacia*, *B. multivorans*, *B. cenocepacia*, *B. vietnamiensis*, *B. ambifaria*, *B. stabilis*, *B. dolosa*, *B. anthina* and *B. pyrrocina* [53].

The AHL QS in the BCC group consists of *luxI/R* homologs known as *cepI* and *cepR*. CepI synthesizes two AHL signals, the C8-HSL (*N*-octanoyl-L-homoserine lactone) and C6-HSL (*N*-hexanoyl-L-homoserine lactone) in greater and lesser amounts respectively. CepR binds and consequently responds to the abundant cognate C8-HSL [53].

The *cepI* and *cepR* genes are divergently transcribed and the intergenic region most commonly contains an *rsaM* homolog. This kind of arrangement is termed as M1 (*R←M→I→*) [31]. The intergenic gene *rsaM* has previously been shown to be a negative regulator of the AHL QS system in *P. fuscovaginae* [35]. Another QS system, called *cciI/R*, has recently been identified in *B. cenocepacia* strains harboring pathogenicity islands as is the case in *B. cenocepacia* K56-2, *B. cenocepacia* J2315 and *B. cenocepacia* MC0-3. This system produces primarily C6-HSL and minor amounts of C8-HSL [54,55]. *cciI* and *cciR* are transcribed in the same direction and accordingly the topology is named as R1. All *B. cenocepacia* members contain a solo or orphan *luxR* homolog named as *cepR2* [29,56]. All QS systems are present on chromosome 2 except in some cases—*bviI/R* of *B. vietnamiensis* (X5 topology) and *luxI/rsaM* of *B. ambifaria*. QS topologies in BCC members are shown in Table 4.

**Figure 2:**
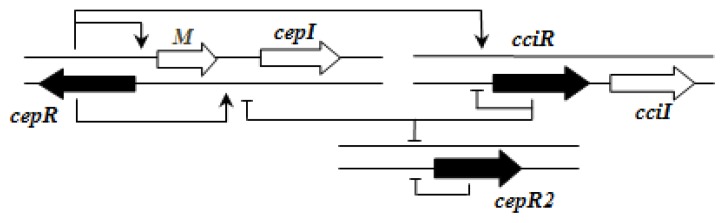
QS regulatory circuits in *Burkholderia cenocepacia* J2315 [54].

Importantly, the QS systems in *B. cenocepacia* are interrelated with each other; CepR regulates *cepI* and *cciIR* genes, while CciR negatively regulates the expression of *cepI* thus allowing for a negative regulatory feedback loop on the *cepI/R* system [55]. The solo/orphan *cepR2* which lacks the associated cognate *luxI*-AHL synthase gene, negatively regulates itself and is also negatively regulated by CciR [57]. Figure 2 shows an example of the regulatory pattern observed in BCC members.

**Figure 3:**
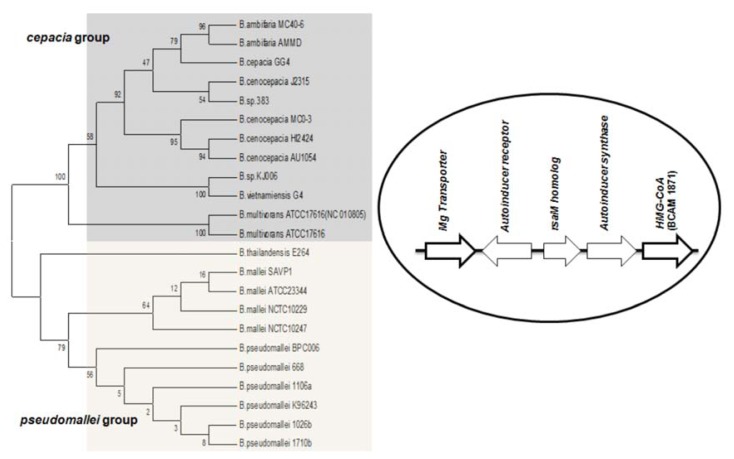
Cladogram of the orthologs of ORF BCAM1871, encoding a protein with an HMG-CoA domain in *Burkholderia* spp. The orthologs are well separated into *cepacia* and *pseudomallei* groups. The RMI (*R←M→I→*) motive is flanked by conserved genes on both sides in members of both *cepacia* and *pseudomallei* groups. The numbers on the tree branches indicate bootstrap values (%).

Studies on *B. cenocepacia* have shown that the production of AHL is also influenced by a neighboring gene downstream from *cepI* [56]. This was identified as ORF BCAM1871, it is co-transcribed with *cepI* and is regulated by CepR. Orthologs of the ORF BCAM1871 contain a domain of 3-hydroxy-3-methyl-glutaryl-CoA reductase family (HMG-CoA reductase). Its genomic location is conserved in all BCC members. Previous studies suggest that BCAM1871 alone cannot activate AHL synthesis, but it enhances the rate of AHL production [56]. Apart from the BCC group, the orthologs of ORF BCAM1871 are also conserved in *B. mallei* and *B. pseudomallei*, it flanks downstream to *luxI* homologs, and the corresponding systems are flanked upstream by an Mg transporter gene. (*i.e*., the M1 topology (RMI) is complemented by two conserved flanking genes in these species) (Figure 3).

**Figure 4:**
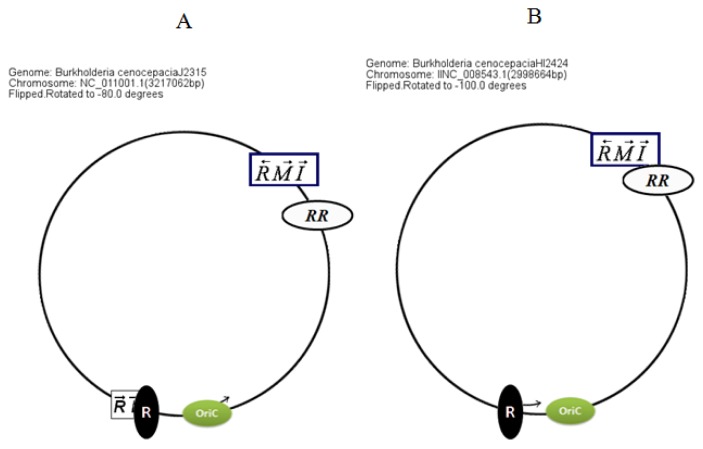
Typical arrangements of QS genes in chromosome II of the *Burkholderia cepacia* complex (BCC*)* group. (**A**) Arrangement of QS genes in the BCC members with *cciR/I* genes (for example *B. cenocepacia* J2315 and *B. cenocepacia* MC0-3); (**B**) Arrangement of QS genes in BCC members having just one pair of *luxR/I* homologs (*cepR/I*) (for example *B. cenocepacia* AU1054, *B. cenocepacia* HI2424, *B. cepacia* GG4, *etc*.). OriC denotes the origin of replication, solo *luxR* genes (*i.e*. those without adjacent I genes) are denoted by black and white ovals, respectively, with the latter indicating two adjacent *luxR* homologues.

Circular map representations reveal that the chromosomal arrangements of the AHL QS genes in BCC members are relatively well conserved with respect to the potential OriC (Figure 4).

### 3.2. *Burkholderia pseudomallei* Group

The *Burkholderia pseudomallei* group consists of *B. mallei* and *B. pseudomallei* subgroups. Both are characterized by multiple AHL QS systems as well as additional *luxR* homologs [58].

#### 3.2.1. Burkholderia mallei

The genome of *B. mallei* contains two *luxI* and four *luxR* homologs arranged as two complete *luxI/R* AHL QS systems and two orphan/solo *luxR* homologs [59]. The complete QS systems in *B. mallei* are called BmaI/R, and their *luxR* homologues *bmaR1* and *bmaR3* respond to signals produced by adjacent *luxI* homolog genes *bmaI1* and *bmaI3*, respectively [60]. BmaI1/R1 shares sequence similarity with BpsI1/R1 in *B. pseudomalle*i, producing and responding to C8-HSL [60]. *bmaI1-bmaR1* are transcribed in opposite orientation to each other and are separated by an intergenic ORF which contains a homolog of *rsaM*. The second LuxI/R homolog pair of *B. mallei* is BmaR3/I3. The *bmaR3-bmaI3* pair is transcribed in the same direction and do not contain ORFs in the intergenic region. BmaI3 produces multiple AHL molecules and includes abundant levels of *N*-3-hydroxy-octanoyl-HSL (3OHC8-HSL). In addition to 3OHC8-HSL, BmaI3 produces *N*-3-hydroxy-hexanoyl-HSL (3OHC6-HSL), and *N*-3-hydroxy-decanoyl-HSL (3OHC10-HSL) in minor amounts. BmaR3 responds to the most abundant product 3OHC8-HSL [59]. The role of orphan/solo LuxR homologs, BmaR4 and BmaR5 are currently unknown. In this genus, all the QS genes are present on chromosome 2.

#### 3.2.2. Burkholderia pseudomallei

The *B. pseudomallei* genome has been identified as having three *luxI/R* pair homologs, namely *bpsI1/R1*, *bpsI2/R2*, and *bpsI3/R3*, and two orphan or solo *luxR* regulator homologs, designated as *bpsR4* and *bpsR5*. With the exception of *bpsR5*, which is present on chromosome 1, most of the QS genes are present on chromosome 2 [61].

The major AHL produced by BpsI1 is C8-HSL(*N*-octanoyl-L-homoserine lactone), whereas BpsI2 and BpsI3 are associated predominantly with *N*-3-hydroxy-octanoyl homoserine lactone (OHC8-HSL) and *N*-3-hydroxy-decanoyl homoserine lactone (OHC10-HSL) respectively [62]. *bpsI1* is divergently transcribed to *bpsR1*, representing the M1 topology, and is separated by an intergenic region which contains an ORF homologous to *rsaM. bpsI2* is transcribed in the same direction as *bpsR2*, representing the M3 topology, and is separated by two to seven genes, one of which is a *rsaM* homolog. *bpsI3* is also transcribed in the same direction as *bpsR3* but is not separated by any of the intergenic ORFs and represents the R1 topology.

The local topological patterns and location of QS genes in members of the *B. pseudomallei* group are shown in Table 5.

**Figure 5:**
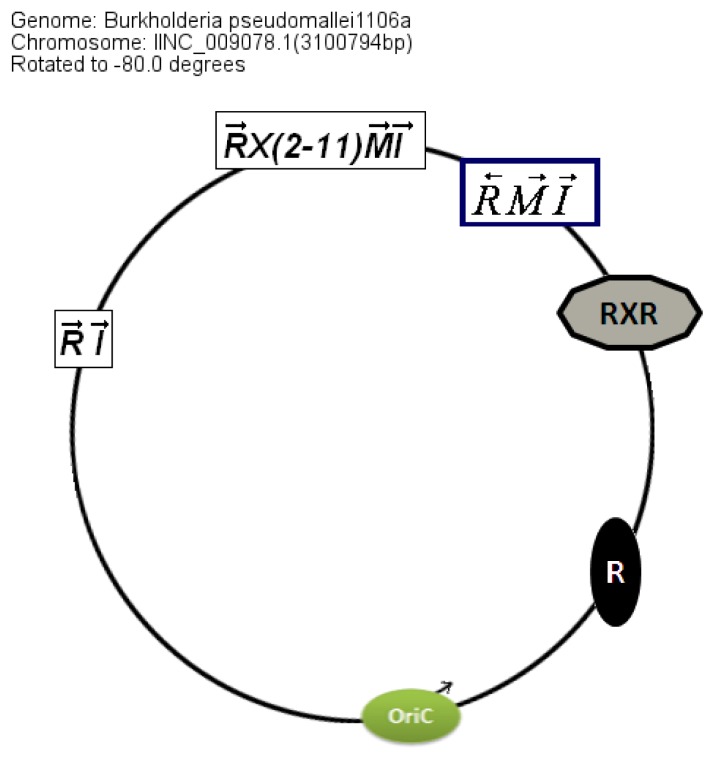
An example of the chromosomal arrangement of QS genes in completely sequenced genome of *B. pseudomallei* strains (for example *B. pseudomallei* 1026b, *B. pseudomallei* 1026a *etc.*)*. RXR*: Two solo *luxR* homologs are separated by a hypothetical gene. The X gene is missing in some *B. pseudomallei*.

The location of the QS genes within the circular chromosome 2 also shows similarities between genomes even though the conservation is stronger in *B. pseudomallei* than in *B. mallei* (Figure 5).

**Figure 6:**
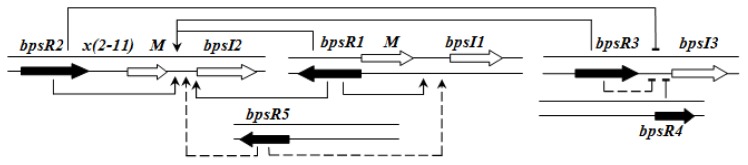
Schematic representation of the complex regulatory circuit in *B. pseudomallei* K92643 [61]. Dashed lines indicate partial regulation.

QS regulation of *B. pseudomallei* is rather complex and hierarchical. BpsR1 regulates *bpsI1* in the presence of a cognate AHL in a positive feedback loop. BpsR5 also partially activates *bpsR1. bpsI2* is constitutively expressed and its expression is enhanced by each of the BpsR—especially by BpsR1 and BpsR3—in the presence of 3-oxo-C8HSL. *bpsI3* is also constitutively expressed but its expression is repressed in the presence of any BpsR protein, although the repression is less in the case of BpsR3 [61] (Figure 6).

### 3.3. Plant-Beneficial and Environmental (PBE) Group

**Figure 7:**
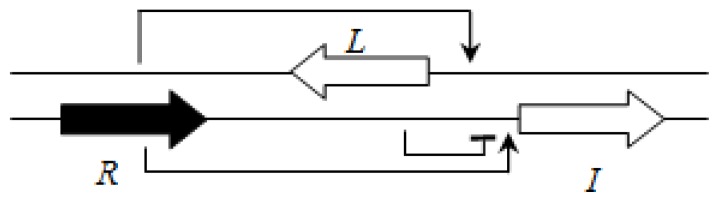
Regulatory circuit of BraR/I system in the plant-beneficial *Burkholderia* group (for example *B. xenovorans* LB400) [63].

This group of the *Burkholderia* genus currently includes 29 non-pathogenic species which are most often associated with plants [63]. The AHL QS systems have been identified only in some members of this group. One system related to the LasI/R system of *P. aeruginosa* is BraI/R, and is highly conserved among all species of this group. The *braR* and *braI* genes are transcribed in the same direction and are under strict negative regulation by the RsaL repressor that is present between the *braR* and *braI* genes [16] (Figure 7). This arrangement is termed the L1 topology (*R→L←I→*).

BraI produces multiple AHLs, including 3-oxo-C6-HSL, 3-oxo-C8-HSL, 3-oxo-C10-HSL, and 3-oxo-C12-HSL and 3-oxo-C14-HSL. BraR responds best to 3-oxo-C14-HSL meaning that is likely to be the cognate AHL for BraI/R systems [63].

Some members of the PBE group (mostly from the *B. xenovorans*, *B. graminis* and *B. phytofirmans* subclade) have an additional AHL system that resembles LuxI/R pairs in other *Burkholderia* species. This additional AHL system is named as XenI2/R2 and produces and responds to 3-hydroxy-C8-HSL (Table 6).

**Figure 8:**
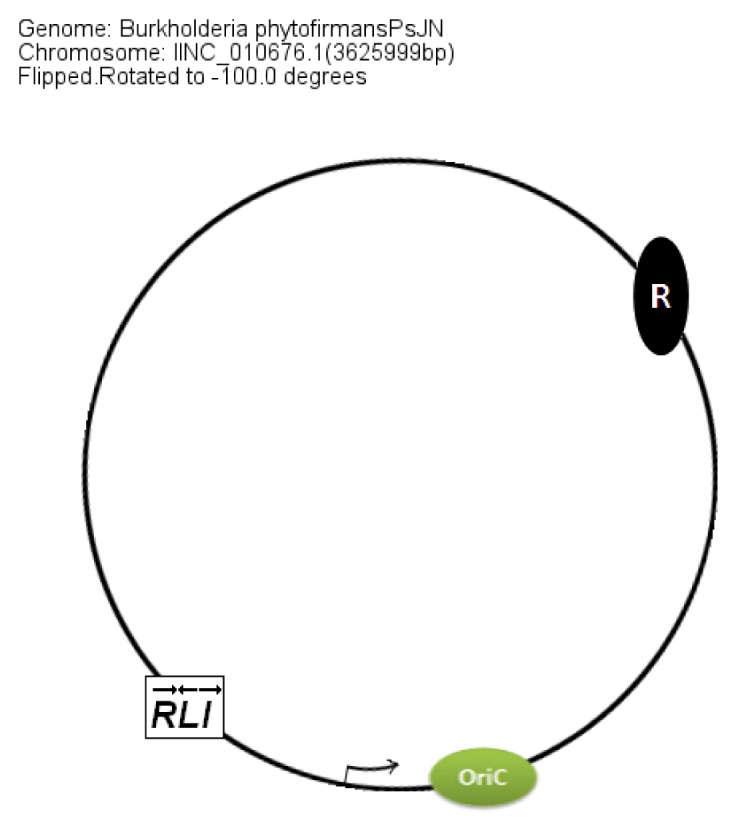
Chromosomal arrangement of QS genes (*braI/R*) and OriC in completely sequenced members of the plant-beneficial and environmental group (for example, *B. phytofirmans* PsJN, *B. xenovorans* LB400, *etc*.).

In members of the PBE group, *braI/R* genes are always present on chromosome 2. The additional QS system XenI2/R2 is present on chromosome 1 and chromosome 3 in *B. phytofirmans* and *B. xenovorans*, respectively. The position of QS genes in the PBE group within the chromosomes was conserved with respect to the *oriC* (Figure 8).

**Figure 9:**
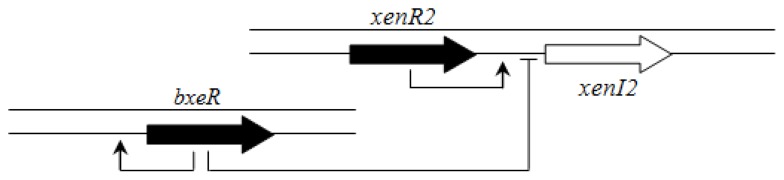
Regulatory circuit of *xenI2/R2* and *bxeR* genes in plant-beneficial and environmental group (for example, *B. xenovorans* LB400) [63].

In contrast to members of the BCC group, there is apparently no hierarchical connection between the BraI/R and XenI2/R2 systems. However, an orphan LuxR homolog called BxeR is present in *B. xenovorans* LB400 and other strains from this cluster (*B. graminis* C4D1M, *B. terricola* LMG30594, *B. phytofirmans* PsJN and *B. phenoliruptrix* AC1100) and negatively regulates *xenI2/R2* [63] (Figure 9).

### 3.4. Other Pathogenic *Burkholderia* spp

There are some human and phytopathogens, including phytopathogenic *B. glumae*, *B. gladioli* and *B. plantarii* that do not cluster with either the BCC or with the *B. pseudomallei* groups. Quorum sensing is known to play an important role in the pathogenicity of these bacteria [64–66]. Typical arrangements are shown in Table 7. *B. glumae* causes disease in rice seedlings and *B. gladioli*, which is phylogenetically close to *B. glumae*, is both a rice pathogen as well as an opportunistic pathogen in humans [65]. Another plant pathogen is *B. plantarii* which also requires the AHL QS system for its pathogenicity [64]. Interestingly, *B. glumae* has different topology of AHL QS genes in two different strains. *luxR* and *luxI* homologs in *B. glumae* BGR1 are divergently transcribed and are separated by an intergenic gene *tofM*, which is an *rsaM* homolog [31,36,67]. In contrast, the LuxR homolog in *B. glumae* ATCC 33617 is 110 amino acids longer and is believed to be non-functional [65]. This *luxR* homolog is also divergently transcribed with respect to its *luxI* homolog but is not separated by any intergenic gene [65]. The topology of *B. gladioli* is similar to *B. glumae* BGR1. In *B. plantarii*, *luxR* and *luxI* homologs are divergently transcribed and are adjacent to each other.

Regulation of the QS system in these species is canonical in that a positive feedback loop is present. The repressive action of TofM in *B. glumae* BGR1 remains unclear and needs to be further studied [36].

## 4. Conclusions

The ability to produce AHL signals is very common in the genus *Burkholderia*. The genes of *Burkholderia* QS systems follow at least 11 typical topological arrangements (10 are common to topological arrangements in other Proteobacteria). Some species, like *B. mallei* and *B. pseudomallei* and certain members of the BCC group, contain multiple AHL QS systems that usually do not operate independently from each other but form a complex hierarchical regulatory network. QS genes in *Burkholderia* are usually located on chromosome 2 where most genes related to virulence and secretion systems are found [14]. This is in agreement with the finding that QS genes regulate pathogenesis and virulence in many species of *Burkholderia*.

The chemical structure of the AHL signals, the local topology of the QS genes and the location of QS systems within the chromosomes show a degree of conservation throughout the entire genus. The fact that some AHL QS systems, which produce OHC8 and OHC10, can be associated with different local topologies in different species possibly indicates that QS topologies evolved before the *Burkholderia* genus separated into the current taxonomical groups. We speculate that the AHL signals appeared before the appearance of the genus and the subsequent rearrangements of the ancestral QS systems lead to the local and chromosomal topologies seen in current species.

The conserved patterns of AHL QS gene neighborhoods is at apparent variance with the general view that gene arrangements in prokaryotes are evolutionarily volatile and may change substantially even on short evolutionary scales when gene sequences diverge minimally [68]. On the other hand, it is generally believed that genome architecture is not a straightforward product of adaptation but rather is determined by an intricate balance between selection pressure, recombination as well as the activity of selfish elements [68]. Seen from this evolutionary perspective we speculate that the appearance of a novel QS system may cause a major change in the lifestyle of a bacterial species, for instance it allows the species to colonize a new nutrient-source. Consequently, there will be selection pressure against losing the new system by genetic rearrangements. On the other hand, continuously present genetic rearrangements may add new elements to the system, such as the regulation of new genes.

A point of interest is the number of AHL QS genes in *Burkholderia*. Galperin and associates argued that the number of bacterial signaling genes is relatively higher in bacteria living in more complex environments, and relatively lower in those living in more sheltered habitats [69,70]. While the original statement referred to two-component systems, a similar general tendency might also be expected with respect to AHL QS systems. The tendency is much less clear, however. Firstly, the number of AHL systems per genome is considerably lower than that of two-component systems. Secondly, the number of AHL systems and solo *luxR* genes is the highest in *Rhizobia* and the *pseudomallei* group of *Burkholderia*, but the other two clades of *Burkholderia* have a low to average number of QS genes. As such, we speculate that the number of AHL genes can be better understood in terms of precise molecular mechanisms connected with the lifestyle of QS bacteria rather than in terms of niche characteristics. It must be noted however that *Burkholderia* also contain at least one other QS system which utilizes another type of signaling molecule which is called Burkholderia Diffusible Signaling Factor (BDSF) [71–73]. The newly identified QS system, just like the AHL system, is very widespread among the *Burkholderia* genus [74,75]. In addition, further unidentified QS systems might exist in the genus; consequently, the mechanism of QS signaling is not yet fully understood in *Burkholderia*.

## Supplementary Information



## Figures and Tables

**Table 1:** Chemical structure of *N*-acylated homoserine lactone (AHL) signals used by LuxR in the known species of the genus *Burkholderia.*

Symbol	Structure	Symbol	Structure
*N*-hexanoyl-*L-*Homoserine lactone**(C6-HSL)**	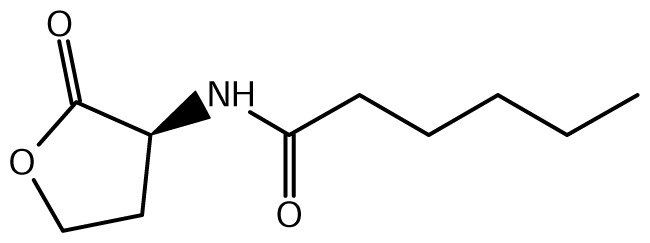	*N*-decanoyl-*L-*Homoserine lactone (**C10-HSL**)	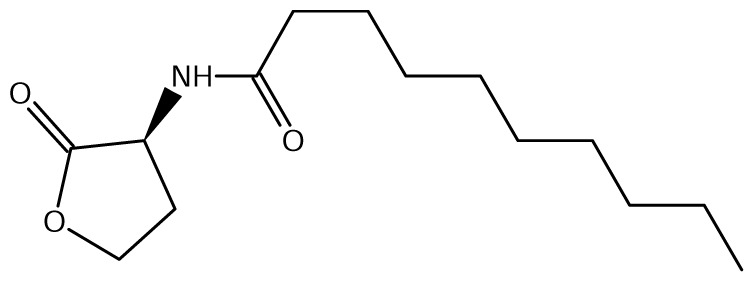
*N*-octanoyl-*L-*Homoserine lactone (**C8-HSL**)	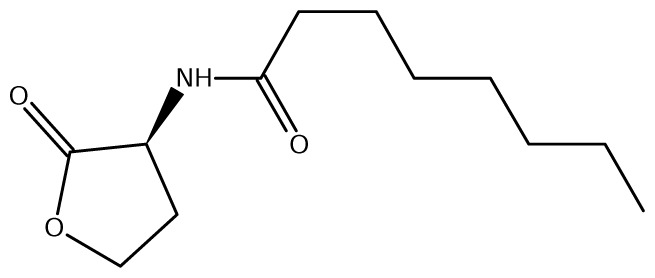	*N*-3-hydroxydecanoyl Homoserine lactone **(OHC10-HSL)**	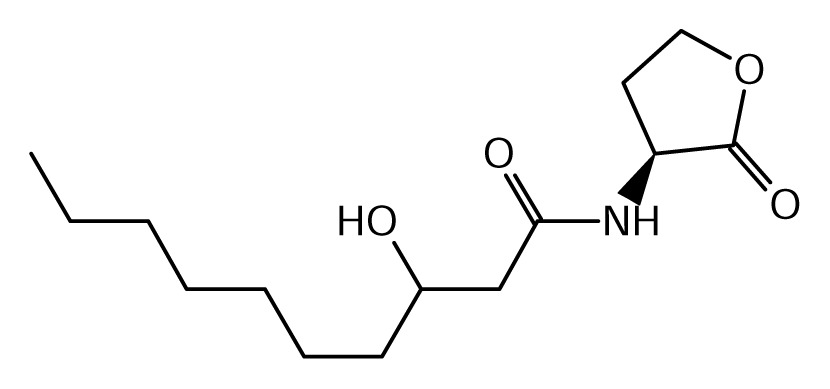
*N*-3-hydroxyoctanoyl Homoserine lactone **(OHC8-HSL)**	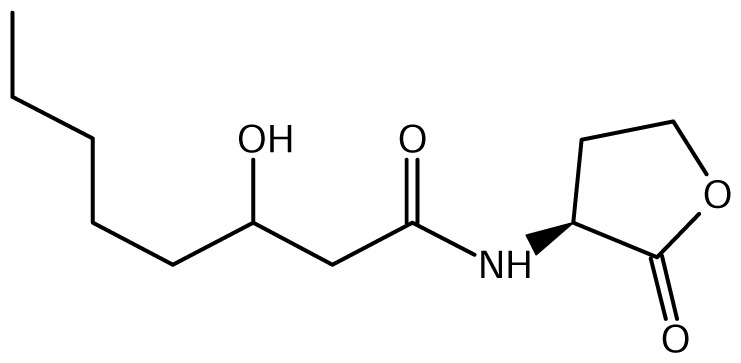	*N*-3-oxotetradecanoyl- *L-*homoserine lactone (**OC14-HSL**)	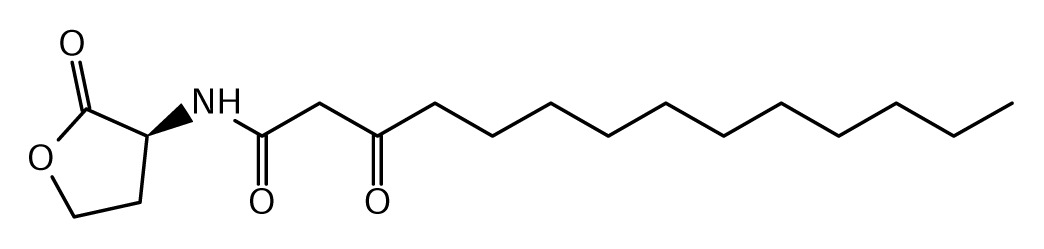

**Table 2:** Typical local topologies of AHL-driven quorum sensing circuits in *Burkholderia*.

ID	Gene topology	Occurrence in *Burkholderia*			

		BCC group	*B. pseudomallei* group	Plant-beneficial/environmental group	Other pathogenic *Burkholderia* species
RI topologies					

**R1**	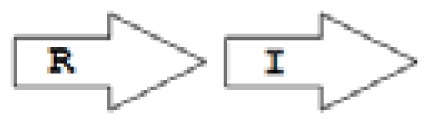	3	17	4	0
**R2**	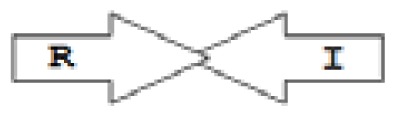	0	0	0	1
**R3**	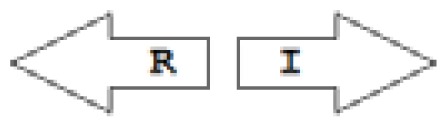	8	2	0	3

RXI topololgies					

**L1**	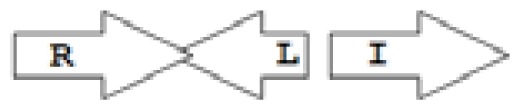	0	0	0	13
**M1**	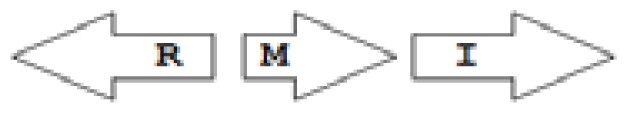	24	46	0	3
**M3**	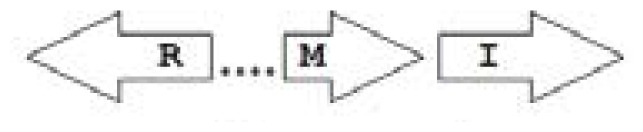	0	30	0	0
**M31**	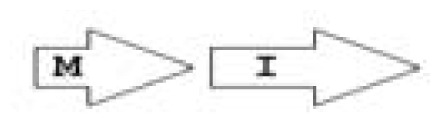	5	5	0	0
**M4**	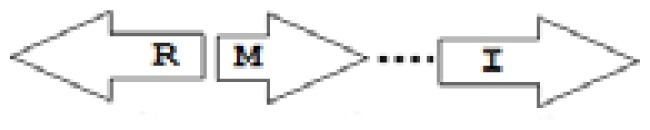	1	0	0	0
**X3**	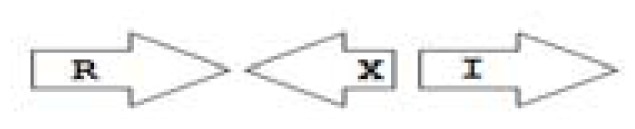	0	24	1	0
**X5**	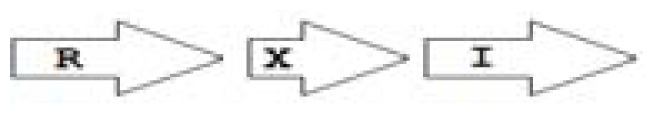	1	0	0	0
**X6**	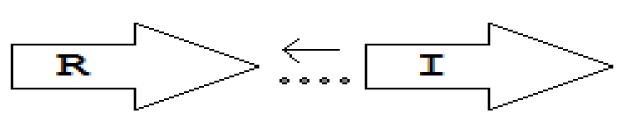	0	1	0	0

**Table 3:** Correlation between the chemical structure and the local gene arrangements in *Burkholderia*.

Symbol	Occurrences in *Burkholderia*			
	BCC group	*B. pseudomallei* group	Plant-beneficial/environmental group	Other pathogenic *Burkholderia* species
**C6-HSL**	**2** (R1)	**0**	0	**0**
**C8-HSL**	**14** (M1)	**13** (M1)	0	**2** (M1: *B. glumae* and R3: *B. plantarii*)
**OHC8-HSL**	0	**11** (R1 and X3: *B. mallei*; R1: *B. thailandensis* and M3 of *B. pseudomallei*)	**3** (R1)	0
**C10-HSL**	**1** (X5)	0	0	0
**OHC10-HSL**	0	**5** (R1 of *B. pseudomallei* and M3 of *B. thailandensis*)	0	0
**OC14-HSL**	0	0	**9** (L1)	0

**Table 4:** Quorum sensing (QS) topologies in the *Burkholderia cepacia* complex.

Species	QS system	ID	Gene Topology	Chr.	Major AHL	Comments
***B. ambifaria***	*bafR/*−*/bafI*	M1	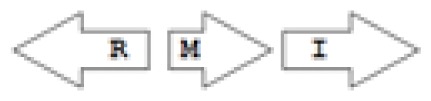	Chr2	C8	Present in all *B. ambifaria* strains
	−*/* −	M31	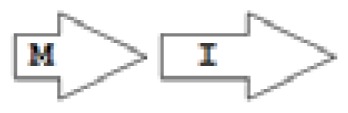	Chr3		Present in some strains such as *B. ambifaria* AMMD, *B.ambifaria* MC40-6
	−*/*−	R1	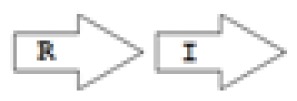	NA		*B. ambifaria* IOP40
***B. cenocepacia***	*cepR/*−*/cepI*	M1	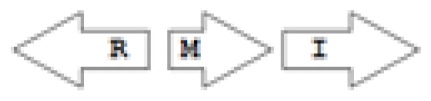	Chr2	C8	Present in all *B. cenocepacia* strains
	*cciR/cciI*	R1	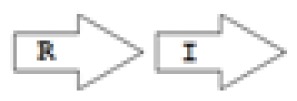	Chr2	C6	Present in some strains such as *B. cenocepacia* K56-2, *B. cenocepacia* J2315 and *B. cenocepacia* MC0-3
***B. multivorans***	*bmuR/*−*/bmuI*	M1	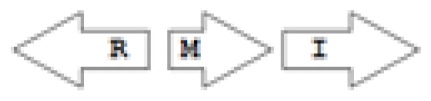	Chr2	C8	*B. multivorans ATCC 17616*
B. multivorans ATCC 17616*	*sdiA/*−*/bmuI*	M1	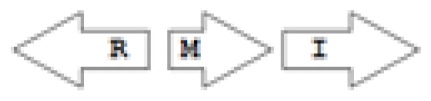	Chr2	C8	B. multivorans ATCC 17616*
***B.vietnamiensis***	*cepR/*−*/cepI*	M1	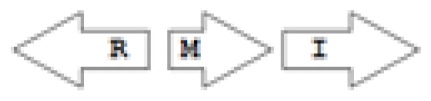	Chr2	C8	*B. vietnamiensis* G4
	*bviR/*−*/bviI*	X5	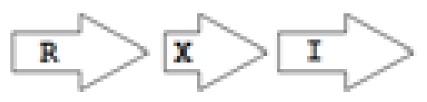	Chr3	C10	*B. vietnamiensis* G4
	−*/* −	R2	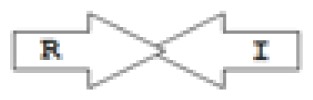	NA		Present in *B. vietnamiensis* C2822, *B. vietnamiensis* G4 etc.
***B. cepacia***	*cepI/cepR*	R2	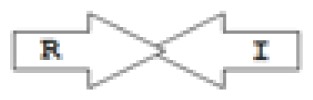	NA		Present in *B. cepacia* ATCC 25416, *B. cepacia* DBO1, *B. cepacia* K56-2

* same strain but different bioproject.

**Table 5:** QS topologies in the *Burkholderia pseudomallei* group.

Species	QS system	ID	Gene topology	Chr.	Major AHL	Comments
***B. mallei***	*bmaR3/bmaI3*	R1	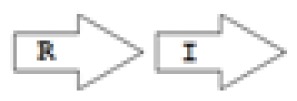	Chr2	OCH8	*B. mallei* ATCC 23344
	*bmaR1/*−*/bmaI1*	M1	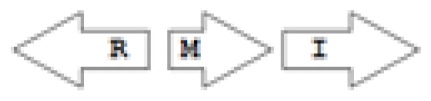	Chr2	C8	Present in all *B. mallei* strains
	*bmaR3/*−*/bmaI3*	X3	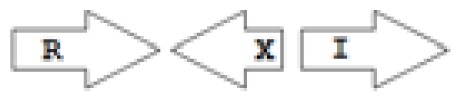	Chr2	OCH8	Present in some strains such as *B. mallei* NCTC 10229 and *B. mallei* NCTC 10247, *B. mallei* FMH *etc*.
***B. pseudomallei***	*bpsR3/bpsI3*	R1	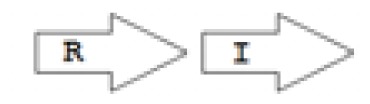	Chr2	OHC10	Present in all *B. pseudomallei* strains
	*bpsR1/*−*/bpsI1*	M1	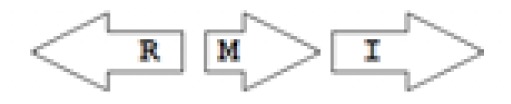	Chr2	C8	Present in all *B. pseudomallei* strains
	*bpsR2/*−*/bpsI2*	M3	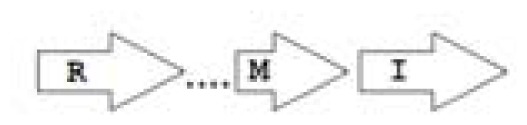	Chr2	OHC8	Present in all *B. pseudomallei* strains
	−*/* −*/* −	X3	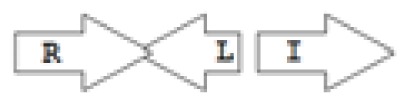	Chr2		Present in some strains such as *B. pseudomallei* 1026b, *B. pseudomallei* MSHR346, *B. pseudomallei* 112 *etc*.
	*bpsI/bpsR*	R2	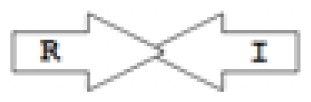	NA		*B. pseudomallei* 844
***B. thailandensis***	*btaR3/btaI3*	R1	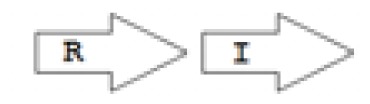	Chr2	OHC8	Present in some strains of *B. thailandensis*
	*btaR1/*−*/btaI1*	M1	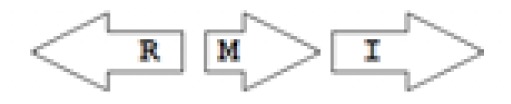	Chr2	C8	Present in all *B. thailandensis* strains
	*btaR2/*−*/btaI2*	M3	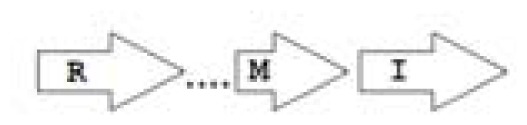	Chr2	OHC8, OHC10	Present in all *B. thailandensis* strains
	−*/*−*/*−	X3	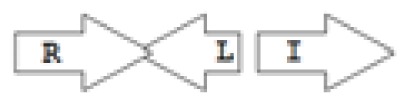	NA		Present in some strains such as *B. thailandensis* Bt4, *B. thailandensis* TXDOH

**Table 6:** QS topologies in plant-beneficial and environmental *Burkholderia* sps.

Species	QS system	ID	Gene topology	Chr. No.	Major AHL	Comments
**Plantbeneficial*Burkholderia*sps.**	*braR/rsaL/braI*	L1	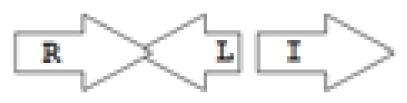	Chr2	**OC14**	All plant-beneficial strains have this system. Ex: *B. phymatum STM815*, *B. phytofirmans PsJN*, *B. xenovorans LB400*, *B. graminis C4D1M*, *B. unamae etc*.
**Plantbeneficial*Burkholderia*sps.**	*xenR2/xenI2*	R1	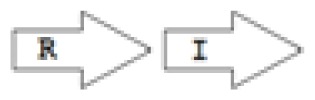	Chr1 (*B. phytofirmans PsJN)*; Chr3 (*B. xenovorans* LB400)	OHC8	Present in some plantbeneficial strains. Ex: *B. phytofirmans PsJN*, *B. xenovorans LB400*, *B. graminis C4D1M*

**Table 7:** QS topologies in plant pathogenic *Burkholderia* sps.

Species	QS system	ID	Gene topology	Chr. No.	Major AHL	Comments
***B. glumae***	*tofR/tofM/tofI*	M1	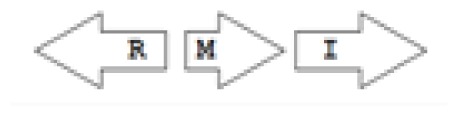	Chr 2	C8	*B. glumae BGR1*, *B. glumae 336gr-1*
	*tofR/tofI*	R3	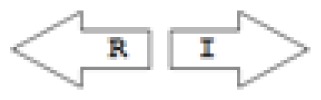	NA	Non functional	*B. glumae* ATCC33617 and *B. glumae* AU6208
***B. gladioli***	−*/*−*/*−	M1	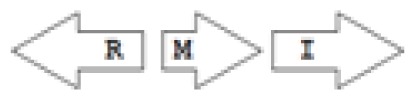	Chr2		*B. gladioli* BSR3
	−*/*−*/*−	M1	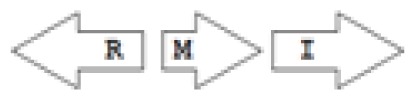	plasmid		*B. gladioli* BSR3
***B. plantarii***	*plaR/plaI*	R3	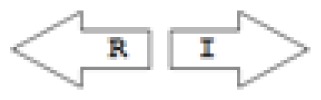	NA	C8	*B. plantarii* ATCC43733
