# Supplementary material for: The Organization of the Quorum Sensing luxI/R Family Genes in Burkholderia
Source: Int J Mol Sci. 2013 Jul 2;14(7):13727–47. doi: 10.3390/ijms140713727 (PMC3742214; doi:10.3390/ijms140713727)
Supplement: Supplementary file 1 [file ijms-14-13727-s001.pdf]

Supplementary Information

Data

Protein fasta sequences of 35 complete genomes and 57 draft genomes containing 30323 contigs and 266 scaffolds were downloaded from NCBI FTP site ftp://ftp.ncbi.nih.gov/genomes/Bacteria/ and ftp://ftp.ncbi.nih.gov/genomes/Bacteria_DRAFT/ respectively. We also included 16585 individual Genbank entries from NCBI RefSeq database (http://www.ncbi.nlm.nih.gov/RefSeq/).

Methods

1. QS Genes Detection

QS genes were determined using Hidden Markov Model (HMM) recognizers build with the HMMER programs HMMER 3.0, http://hmmer.janelia.org/), based on four core set of protein sequences of LuxR, LuxI, RsaL and RsaM proteins (see Table S1 below). These core sets were downloaded from UNIPROT database (http://www.uniprot.org). The CLUSTAL program (accessed via the EBI Webportal, http://www.ebi.ac.uk/Tools/msa/clustalw2/) was used for constructing the multiple sequence alignments which were then processed by the HMMBUILD program to build HMM recognizers that were in turn used to scan the protein sequence data. Hits stronger than E- value of $1e^{-10}$ were taken as potential homologues of QS genes. These were manually checked and the local topologies were assigned according to the arrangement of QS genes.

Table S1. List of protein sequence sets used for building HMM recognizers.

luxI homologues
YP_002649215.1, YP_003261728.1, YP_003262850.1, YP_003296640.1, YP_003331715.1, YP_003608088.1, YP_004230809.1, YP_003366470.1, YP_004012993.1, YP_004106681.1, YP_004106954.1, YP_004108425.1, YP_003910269.1, YP_003520250.1, YP_003530770.1, YP_003538486.1, YP_003546445.1, YP_003558209.1, YP_003566926.1, YP_003568278.1, YP_003576501.1, YP_003729883.1, YP_003930460.1, YP_003734012.1, YP_003749682.1, YP_003750860.1, YP_003744153.1, YP_003740503.1, YP_003740954.1, YP_003847234.1, YP_003885141.1, YP_004088230.1, YP_004115279.1, NP_521405.1, NP_522340.1, NP_767703.1, NP_106262.1, NP_106661.1, NP_109412.1, NP_385945.1, YP_002965845.1, YP_002966879.1, YP_002346031.1, YP_002347420.1, YP_002426405.1, YP_428477.1, YP_105963.1, YP_106161.1, YP_110894.1, YP_111576.1, YP_001005892.1, YP_554693.1, YP_555669.1, YP_165635.1, YP_167511.1, NP_669050.1, NP_670673.1, YP_528965.1, YP_234707.1, NP_250123.1, NP_252166.1, YP_002232872.1, YP_002234481.1, YP_768958.1, YP_048233.1, NP_793636.1, YP_789671.1, YP_791820.1, NP_903761.1, NP_993604.1, NP_994737.1, YP_674865.1, YP_371808.1, YP_001114940.1, YP_001117676.1, YP_439001.1, YP_439708.1, YP_273860.1, YP_508562.1, YP_071011.1, YP_071751.1, YP_206882.1, YP_914595.1, YP_002551489.1, YP_002549360.1, YP_002541324.1, YP_659946.1, YP_317245.1, YP_776005.1, YP_617566.1, YP_617628.1, YP_838353.1, YP_623506.1, YP_470411.1, YP_473057.1, YP_001024425.1, YP_001025818.1, YP_001077901.1, YP_001078152.1, YP_989942.1, YP_001062290.1, YP_001063210.1, YP_335777.1, YP_337633.1, YP_972130.1, YP_484039.1, YP_486927.1, YP_567542.1, YP_569311.1, YP_530592.1, YP_531903.1, YP_781244.1, YP_001231849.1, YP_001604809.1, YP_001606209.1, YP_001399709.1, YP_001400525.1, YP_001220569.1, YP_001241094.1, YP_001242901.1, YP_001075256.1, YP_001076162.1, YP_002537871.1, YP_001327237.1, YP_453964.1, YP_681952.1, YP_650194.1, YP_651865.1, YP_647981.1, YP_649109.1, YP_002220095.1, YP_855089.1,

YP_001161918.1, YP_001163229.1, YP_001347034.1, YP_001349251.1, YP_001143471.1, YP_001583944.1, YP_001860597.1, YP_001811255.1, YP_001531662.1, YP_001534185.1, YP_001761364.1, YP_001476305.1, YP_001888022.1, YP_001893789.1, YP_001083198.1, YP_001844795.1, YP_001777918.1, YP_001779189.1, YP_001641952.1, YP_001772211.1, YP_001758390.1, YP_001776814.1, YP_001783295.1, YP_002158590.1, YP_001948920.1, YP_002423669.1, YP_001927659.1, YP_001203094.1, YP_002128524.1, YP_002976728.1, YP_001989358.1, YP_001991324.1, YP_002282165.1, YP_002495630.1, YP_002496260.1, YP_002497058.1, YP_001906897.1, YP_001908005.1, YP_001832057.1, YP_002826208.1, YP_002317565.1, YP_001979200.1, YP_001985290.1, YP_001719546.1, YP_001720402.1, YP_001873009.1, YP_001873806.1, YP_002265246.1, YP_002327281.1, YP_002439140.1, YP_002441565.1, YP_002923740.1, YP_003019698.1, YP_003002473.1, YP_002955226.1, YP_002909043.1, YP_002934276.1, YP_002360442.1, YP_002947663.1, YP_003964946.1, YP_003941574.1, YP_001603070.1, YP_003070966.1, YP_001715479.1, YP_004144716.1, YP_004145051.1, NP_945673.1
LuxR homologues
YP_002649216.1, YP_003261727.1, YP_003262848.1, YP_003296639.1, YP_003331714.1, YP_003608086.1, YP_004230807.1, YP_003366469.1, YP_004012994.1, YP_004106680.1, YP_004106955.1, YP_004108424.1, YP_003910271.1, YP_003520251.1, YP_003530769.1, YP_003538485.1, YP_003546444.1, YP_003558208.1, YP_003566925.1, YP_003568279.1, YP_003576500.1, YP_003729882.1, YP_003930459.1, YP_003734010.1, YP_003749681.1, YP_003750859.1, YP_003744152.1, YP_003740504.1, YP_003740953.1, YP_003847232.1, YP_003885142.1, YP_004088229.1, YP_004115278.1, NP_521406.1, NP_522339.1, NP_767702.1, NP_106261.1, NP_106660.1, NP_109411.1, NP_385944.1, YP_002965846.1, YP_002966880.1, YP_002346032.1, YP_002347421.1, YP_002426403.1, YP_428476.1, YP_105961.1, YP_106160.1, YP_110896.1, YP_111575.1, YP_001005891.1, YP_554691.1, YP_555670.1, YP_165634.1, YP_167510.1, NP_669049.1, NP_670674.1, YP_528967.1, YP_234708.1, NP_250121.1, NP_252167.1, YP_002232873.1, YP_002234479.1, YP_768957.1, YP_048234.1, NP_793635.1, YP_789670.1, YP_791822.1, NP_903760.1, NP_993605.1, NP_994736.1, YP_674864.1, YP_371810.1, YP_001114942.1, YP_001117674.1, YP_439002.1, YP_439706.1, YP_273861.1, YP_508561.1, YP_071012.1, YP_071752.1, YP_206883.1, YP_914594.1, YP_002551488.1, YP_002549361.1, YP_002541325.1, YP_659944.1, YP_317246.1, YP_776003.1, YP_617565.1, YP_617627.1, YP_838351.1, YP_623508.1, YP_470410.1, YP_473056.1, YP_001024423.1, YP_001025820.1, YP_001077903.1, YP_001078154.1, YP_989940.1, YP_001062292.1, YP_001063209.1, YP_335776.1, YP_337635.1, YP_972129.1, YP_484040.1, YP_486928.1, YP_567541.1, YP_569310.1, YP_530593.1, YP_531902.1, YP_781245.1, YP_001231850.1, YP_001604810.1, YP_001606210.1, YP_001399708.1, YP_001400524.1, YP_001220570.1, YP_001241092.1, YP_001242900.1, YP_001075258.1, YP_001076161.1, YP_002537872.1, YP_001327236.1, YP_453965.1, YP_681951.1, YP_650193.1, YP_651866.1, YP_647982.1, YP_649110.1, YP_002220093.1, YP_855090.1, YP_001161917.1, YP_001163230.1, YP_001347033.1, YP_001349253.1, YP_001143472.1, YP_001583946.1, YP_001860599.1, YP_001811253.1, YP_001531661.1, YP_001534186.1, YP_001761363.1, YP_001476304.1, YP_001888024.1, YP_001893790.1, YP_001083200.1, YP_001844797.1, YP_001777917.1, YP_001779191.1, YP_001641953.1, YP_001772212.1, YP_001758389.1, YP_001776815.1, YP_001783296.1, YP_002158591.1, YP_001948918.1, YP_002423670.1, YP_001927660.1, YP_001203095.1, YP_002128523.1, YP_002976727.1, YP_001989359.1, YP_001991323.1, YP_002282164.1, YP_002495629.1, YP_002496262.1, YP_002497059.1, YP_001906896.1, YP_001908006.1, YP_001832058.1, YP_002826207.1, YP_002317567.1, YP_001979199.1, YP_001985289.1, YP_001719545.1, YP_001720401.1, YP_001873010.1, YP_001873807.1, YP_002265247.1, YP_002327279.1, YP_002439139.1,

YP_002441567.1, YP_002923741.1, YP_003019697.1, YP_003002472.1, YP_002955225.1, YP_002909041.1, YP_002934275.1, YP_002360441.1, YP_002947664.1, YP_003964947.1, YP_003941575.1, YP_001603072.1, YP_003070967.1, YP_001715477.1, YP_004144717.1, YP_004145052.1, NP_945674.1,
RsaL homologue
NP_250122.1, YP_001349252.1, YP_001860598.1, YP_001888023.1, YP_002441566.1, YP_002794907.1, YP_003608087.1, YP_003847233.1, YP_003910270.1, YP_554692.1, YP_791821.1
RsaM homologues:
YP_439707.1, YP_001062653.1, YP_776004.1, YP_001117675.1

2. Chromosomal Arrangement of QS Genes

In order to depict the topological patterns of QS genes visually with respect to OriC, we wrote a Python script, which uses topologies and their coordinates in the genome as input parameters and generates circular genome diagrams. The script scales the coordinates to 2π degrees, in order to draw into a circle. In addition, the coordinates of the OriC were included to serve as a reference point in terms of visualizing the distance of topologies. OriC was calculated from GenSkew tool (http://genskew.csb.univie.ac.at/) which computes and plots nucleotide skew data. The region with minimum GC skew is generally considered to be site of origin of replication. Since, coordinates of *luxI*, *luxR*, *rsaL* and *rsaM* are very near to each other and are difficult to distinguish; we therefore chose coordinates of *luxR* genes in each case.

3. Evolutionary Relationships

Cladograms for selected sequence groups were built using the guide tree of the CLUSTAL program and visualized using the MEGA5 program package installed from http://www.megasoftware.net. The numerical values at each branch indicate the bootstrap values (%).

The pictorial representation of the standard procedure carried for annotation of QS genes is shown in Figure S1.

Figure S1. Flowchart representation of the Automated Genome Annotation Pipeline.

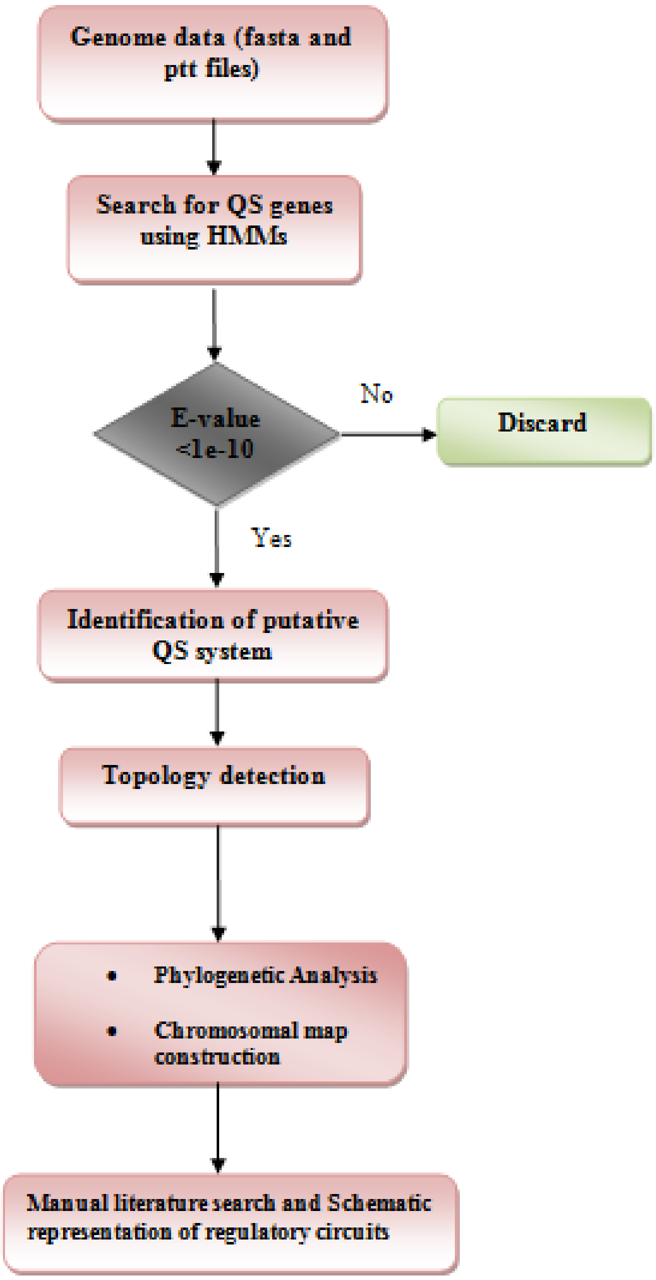

The cladogram of all the LuxI proteins present in *Burkholderia* genomes is shown in supplementary file 2 (**Figure S2.** Cladogram of LuxI proteins present in *Burkholderia* genomes).

© 2013 by the authors; licensee MDPI, Basel, Switzerland. This article is an open access article distributed under the terms and conditions of the Creative Commons Attribution license (http://creativecommons.org/licenses/by/3.0/).
